# Communication between Corneal Epithelial Cells and Trigeminal Neurons Is Facilitated by Purinergic (P2) and Glutamatergic Receptors

**DOI:** 10.1371/journal.pone.0044574

**Published:** 2012-09-07

**Authors:** Duane J. Oswald, Albert Lee, Monique Trinidad, Cheryl Chi, Ruiyi Ren, Celeste B. Rich, Vickery Trinkaus-Randall

**Affiliations:** 1 Departments of Biochemistry and Ophthalmology, Boston University School of Medicine, Boston, Massachusetts, United States of America; 2 Boston University School of Medicine, Boston, Massachusetts, United States of America; University of California, Berkeley, United States of America

## Abstract

Previously, we demonstrated that nucleotides released upon mechanical injury to corneal epithelium activate purinergic (P2) receptors resulting in mobilization of a Ca^2+^ wave. However, the tissue is extensively innervated and communication between epithelium and neurons is critical and not well understood. Therefore, we developed a co-culture of primary trigeminal neurons and human corneal limbal epithelial cells. We demonstrated that trigeminal neurons expressed a repertoire of P2Yand P2X receptor transcripts and responded to P2 agonists in a concentration-dependent manner. Mechanical injuries to epithelia in the co-cultures elicited a Ca^2+^ wave that mobilized to neurons and was attenuated by Apyrase, an ectonucleotidase. To elucidate the role of factors released from each cell type, epithelial and neuronal cells were cultured, injured, and the wound media from one cell type was collected and added to the other cell type. Epithelial wound media generated a rapid Ca^2+^ mobilization in neuronal cells that was abrogated in the presence of Apyrase, while neuronal wound media elicited a complex response in epithelial cells. The rapid Ca^2+^ mobilization was detected, which was abrogated with Apyrase, but it was followed by Ca^2+^ waves that occurred in cell clusters. When neuronal wound media was preincubated with a cocktail of N-methyl-D-aspartate (NMDA) receptor inhibitors, the secondary response in epithelia was diminished. Glutamate was detected in the neuronal wound media and epithelial expression of NMDA receptor subunit transcripts was demonstrated. Our results indicate that corneal epithelia and neurons communicate via purinergic and NMDA receptors that mediate the wound response in a highly orchestrated manner.

## Introduction

When epithelium is injured, the cells surrounding the wound migrate into the wound bed to re-establish the integrity of the tissue. The repair requires a controlled and collaborative system of communication between epithelial and neuronal cells to resynthesize the damaged matrix [1], [2], migrate into the wound bed [3]–[5], and return the epithelium to its original architecture. Previous studies have shown that damage to corneal epithelial cells results in release of nucleotides into the extracellular space at the wound site [6]–[8]. The nucleotide agonists bind purinergic (P2) receptors in a dose-dependent manner [6], [9], [10], activate the receptor and produce an increase in intracellular Ca^2+^ resulting in the mobilization of a wave to adjacent cells [6], [11]. Additional nucleotides may diffuse from the activated cells through hemichannels opening into the extracellular space and act in a paracrine manner by binding purinergic receptors on adjacent cells [12]–[15].

The purinergic family is subdivided into two major sub-groups: ionotropic P2X receptors and metabotropic P2Y receptors. P2X receptors are further divided into seven subtypes, P2X_1–7_. Upon binding of ATP, P2X receptors produce cationic selective pores in the extracellular membrane consisting of homo- or heterotrimeric subunits, which are cell-type dependent [16], [17]. P2X receptor activation is thought to be linked with nociception in neurons [18], [19], inflammation [20], [21] and apoptosis [22]. The P2Y receptors are divided into eight subtypes: P2Y_1_, P2Y_2_, P2Y_4_, P2Y_6_, and P2Y_11–14_ and the expression pattern varies between cell types [10], [23]. They are thought to play roles in neuroprotection [24], synaptic transmission modulation [25]–[27] and wound healing [6]–[8]. Recently, we have shown that the P2Y_2_ receptor plays a key role in injury-induced propagation of Ca^2+^ mobilization, wound repair and transactivation of the epidermal growth factor receptor (EGFR) in epithelial cells [28], [29].

The avascular, squamous corneal epithelium is innervated by a dense mesh of sensory processes derived from the ophthalmic branch of the trigeminal nerve [30], [31]. These nerve endings are responsible for nociception, cold, and pressure transmission [32]–[35]. Denervation of the cornea reduces the extent and increases the duration of wound closure [36]–[39]. Trophic factors, such as the neuropeptide substance P [40], released by the trigeminal neurons may participate in this process, but our data indicate that other factors are critical.

In the present study, we demonstrate the P2 receptor expression profile for trigeminal sensory neurons and show that trophic factors released from the trigeminal neurons mediate cell-cell communication in corneal epithelial cells. To investigate cell communication, a co-culture model of primary trigeminal sensory neurons and corneal epithelial cells was developed. Injury to epithelial cells resulted in a release of nucleotides and the mobilization of a Ca^2+^ wave from the epithelium to the neurons, which was attenuated with Apyrase. The addition of epithelial wound media to neuronal cells elicited a rapid on-off Ca^2+^ mobilization that was inhibited with Apyrase. In contrast, the addition of neuronal wound media to epithelia caused a complex response due to the release of both nucleotides and glutamate receptor agonists. The epithelial cells expressed NMDA receptor transcripts and NMDA receptor subunit 1 (NR1) was detected in epithelial cultures. Furthermore, incubation of neuronal wound media with a cocktail of NMDA receptor inhibitors depressed the secondary mobilization of Ca^2+^. We speculate that ATP is released first from epithelial cells and is followed by the release of ATP and glutamate from neuronal processes that activate both purinergic and NMDA receptors on epithelial cells. The novel glutamatergic-purinergic interactions may promote both the wound response and prolonged communication between cells. Together, these results demonstrate a paradigm shift in the mode of communication that occurs between corneal epithelial cells and sensory neurons to initiate a wound healing process.

## Results

### Trigeminal Neuronal Cultures Express P2Y and P2X Receptors and Demonstrate a dose Dependent Response to Purinergic Agonists

**Figure 1:**
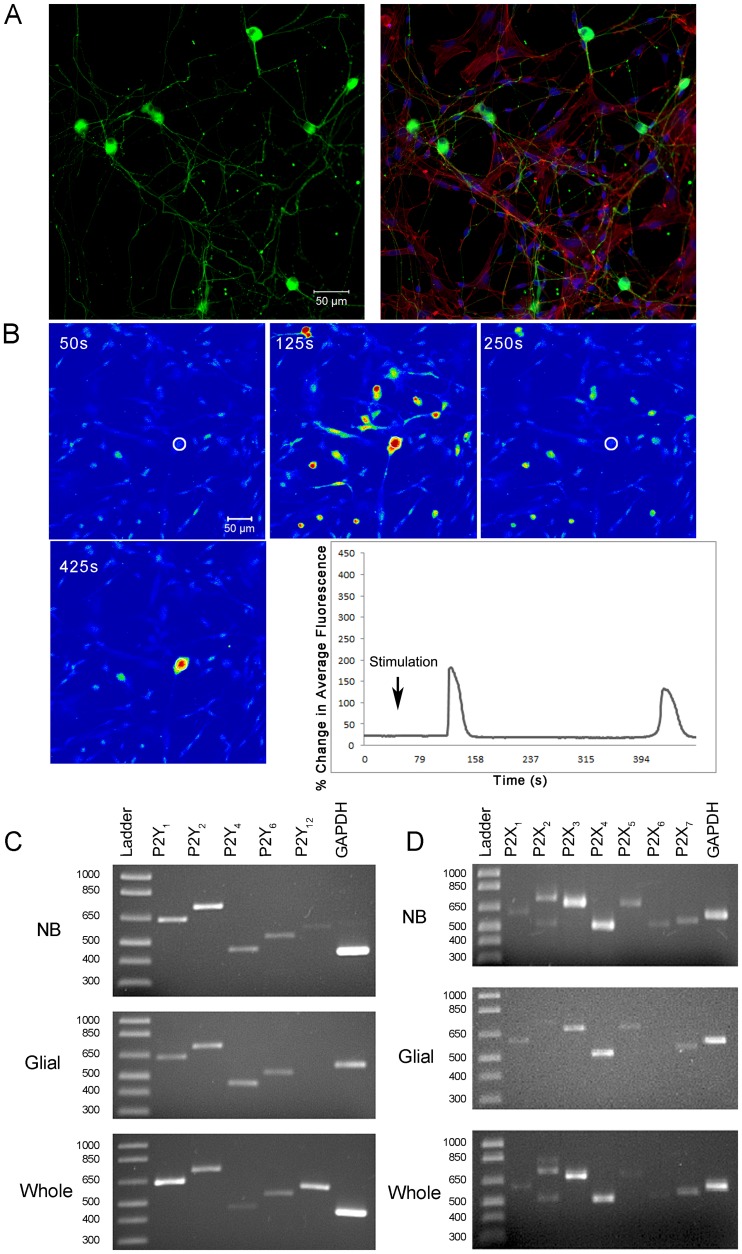
**Primary trigeminal sensory neuron cultures.** **A.** Cells were isolated and cultured for one week in neurobasal medium. Cells were stained with anti-β-tubulin (Tuj1) antibody (green) (left panel) and counter-stained for F-actin with rhodamine phalloidin (red) and for nuclei with To-Pro-3 (blue) (right panel). **B.** Neuronal cells were incubated in 5 µM fluo3-AM for 30 min, washed and imaged in a flow-through apparatus on a Zeiss LSM 510 confocal microscope. Once basal levels were established with HEPES, cells were stimulated a single time with 25 µM ATP (arrow). A series of images from a representative experiment is shown and a graph of a single cell (white circle) is followed over 600 frames and presented as percent change in average fluorescence. The data represent 9 runs from 3 independent cultures. **C.** and **D.** To determine the expression profiles of P2Y and P2X receptors, mRNA was extracted from three independent primary neuronal cultures in neurobasal medium (NB), cultures of glial cells, or whole trigeminal ganglia branch tissue and reverse transcribed using M-MLV reverse transcriptase. 50 ng of cDNA was PCR amplified using rat P2Y or P2X- transcript-specific primers.

The neuronal population was cultured in neurobasal media with B27 to enrich the population of neuronal cells. The sensory neurons grew processes with growth cones at their termini within 24 h; within 48 h, a complex network of fine fibers was detected. The neurons spread out along the glia in the culture and were identified with an antibody directed against ?-tubulin III (Fig. 1A). The neural somata were globular with diameters ranging between 20–30 µm. The glia were identified with glial fibrillary acidic protein (GFAP).

Previously, we showed that the P2Y_2_ receptor is critical for mobilization of a Ca^2+^ wave after injury and in mediating migration of epithelial cells in scratch wound assays [28], [29]. In the present study, we evaluated the neuronal response to the nucleotide-induced stimulation. The cells were stimulated with ATP to determine if the P2 receptors were activated in the neuronal-enriched cultures. A representative response is shown in Fig. 1B. In addition, the response to nucleotides (ATP, ADP, UTP and UDP) was evaluated over a range of concentrations. To ensure that phenol red present in both neurobasal and Dulbecco’s Modified Eagle Medium (DMEM) did not artificially alter the regulation of P2X_1–3_ receptors, experiments were performed in phenol-free custom-made media. For each run, cells were imaged with HEPES buffer alone and an average baseline was obtained. Cells were stimulated and the percent of responders was calculated for each condition [11], [29]. There was a dose-dependent response as the concentration of ATP was increased (5, 10, 25, 50, 100 µM) and at 100 µM ATP over 95% of the neurons displayed an increase in fluorescence. The trend was similar for ADP, UTP and UDP (Table 1). In addition, the maximal percent change in average fluorescence above background was calculated. Five µM ATP induced a 5% increase over HEPES while 100 µM ATP induced a 124% increase. Likewise at 100 µM, there was a similar response with the other agonists **(** Table 1 **)**.

**Table 1:** Cellular response to purinergic agonists.

	% of neurons responding		% change in average fluorescence at 100 µM
Stimulation	1 µM	100 µM	
ATP	50%	>95%	124%
ADP	35.4%	85.8%	120%
UTP	16.7%	97.1%	91%
UDP	57.6%	98.1%	208%

Because the cultures responded to ATP, the expression profile of both P2Y and P2X receptors was identified and compared to that of intact trigeminal cells. While we have shown the expression profile of P2X and P2Y receptors in corneal epithelium [41], the expression profile of P2Y and P2X receptors for neuronal cells was not known in detail nor had it been compared to the in vivo tissue. A partial examination of trigeminal ganglia neurons served as reference [42]. The neuronal enriched cultures expressed the full complement of P2Y receptors, as did the intact trigeminal branch (Fig. 1C). In addition, the neurons expressed the repertoire of P2X receptors (Fig. 1D). In contrast, the glial cultures did not express P2Y_12_, P2X_2_ or P2X_6_. As the epithelial cells express the P2Y receptors (P2Y_1,2,4,6_) and the P2X receptors (P2X _4,5,6,7_) [41], there is redundancy in expression of these receptors in the cornea. Together, these results indicate that the neuronal cells express a complex repertoire of P2Y and P2X receptors.

As the cells responded to ATP and expressed both P2Y and P2X receptors, we asked if there was a sensitivity to divalent cations. In addition external concentrations of calcium have been shown to have an antagonizing effect on ATP binding by the P2X_2_ neuronal receptor, particularly at concentrations above 5 mM [43]. When the cultures were stimulated with 25 µM ATP in HEPES buffer containing a lower concentration of CaCl_2_ (3 mM), the maximum average percent change in fluorescence was measured to be 61.7% and 100% of the total number of neurons responded. When cells were switched to HEPES buffer containing 1.7 mM CaCl_2_, the maximal percent change in average fluorescence dropped to 37%, while 100% of the cells still responded. A further decrease was detected in Ca^2+^-free HEPES buffer where the maximal percent change in average fluorescence decreased to 23.1% with only 71.4% of the cells responding compared to 100% responders in 3 mM CaCl_2_. Monovalent Mg^2+^ has been shown to antagonize P2X_2_,_4_ receptors with an EC_50_ of 2.2 mM [43]. When cells were stimulated with 25 µM ATP in Mg^2+^-free HEPES buffer containing 3 mM CaCl_2_ there was a positive percent change in fluorescence from 61% to 174%. These data indicate that when cells are stimulated or injured in standard HEPES buffer containing Mg^2+^, the external Mg^2+^ reduced the percent change in fluorescence in neuronal cells.

### Role of Gap Junctions/hemichannels in Neuronal Communication

**Figure 2:**
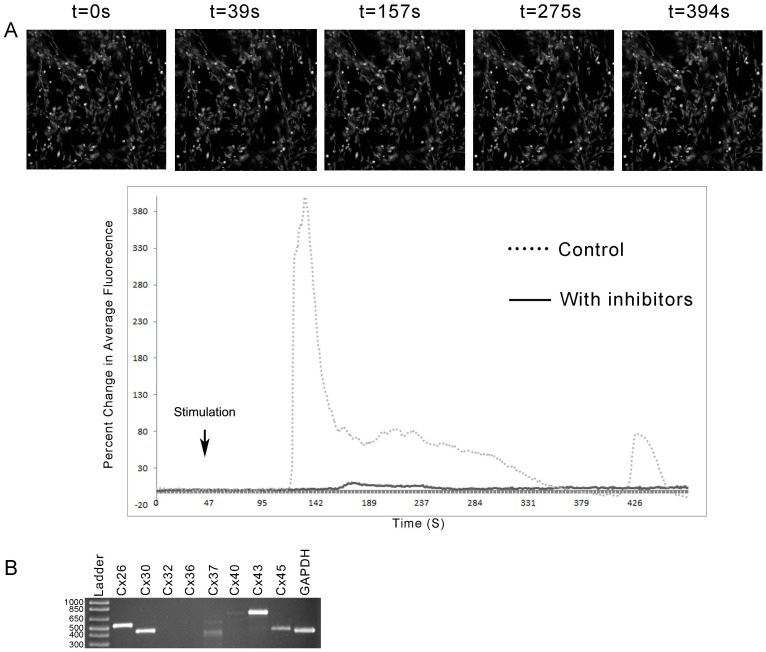
Neuronal cell communication depends on extracellular Ca^2+^ and Connexin/hemichannels. **A.** Neuronal cells were incubated in 5 µM fluo3-AM for 30 min, washed and imaged in a flow-through apparatus on a Zeiss LSM 510 confocal microscope. Cells were incubated with a cocktail of gap junction/hemichannel inhibitors and stimulated a single time with 25 µM ATP (arrow). A series of images from a representative experiment is shown indicating a minimal response. The maximal percent change in average fluorescence was graphed. The dotted line indicates an average of 9 runs of neuronal cultures stimulated with 25 µM ATP without incubation in the presence of inhibitors. **B.** RT-PCR of neuronal extracts showed the expression of Connexin transcripts. The data are representative of a minimum of 3 independent experiments.

Experiments were performed to test if ATP was released into the extracellular space through connexin hemichannels or if cells communicated via gap junctions. When the neuronal cultures were incubated with either 2 mM 1-heptanol or 100 µM meclofenamic acid alone and then stimulated there was no reduction in percent change in fluorescence in response to nucleotides (25 µM ATP) compared to agonist in HEPES buffer. When the neuronal cells were incubated with a cocktail of inhibitors targeting connexin hemichannels (2 mM 1-heptanol, 10 µM CdCl_2_, 7 µM 18a- glycerrhatinic acid (GA) and 100 µM meclofenamic acid) and stimulated with ATP, the response was reduced. There was less than a 20% change in average maximal fluorescence when cells were treated with the inhibitors (N = 9 for each) (Fig. 2A). While RT-PCR of neuronal extracts revealed the presence of connexin transcripts, the response indicates that communication may be more complicated (Fig. 2B). These indicate that the communication did not occur through simple gap junctions as the response was not reduced until cells were incubated with a cocktail of inhibitors.

**Figure 3:**
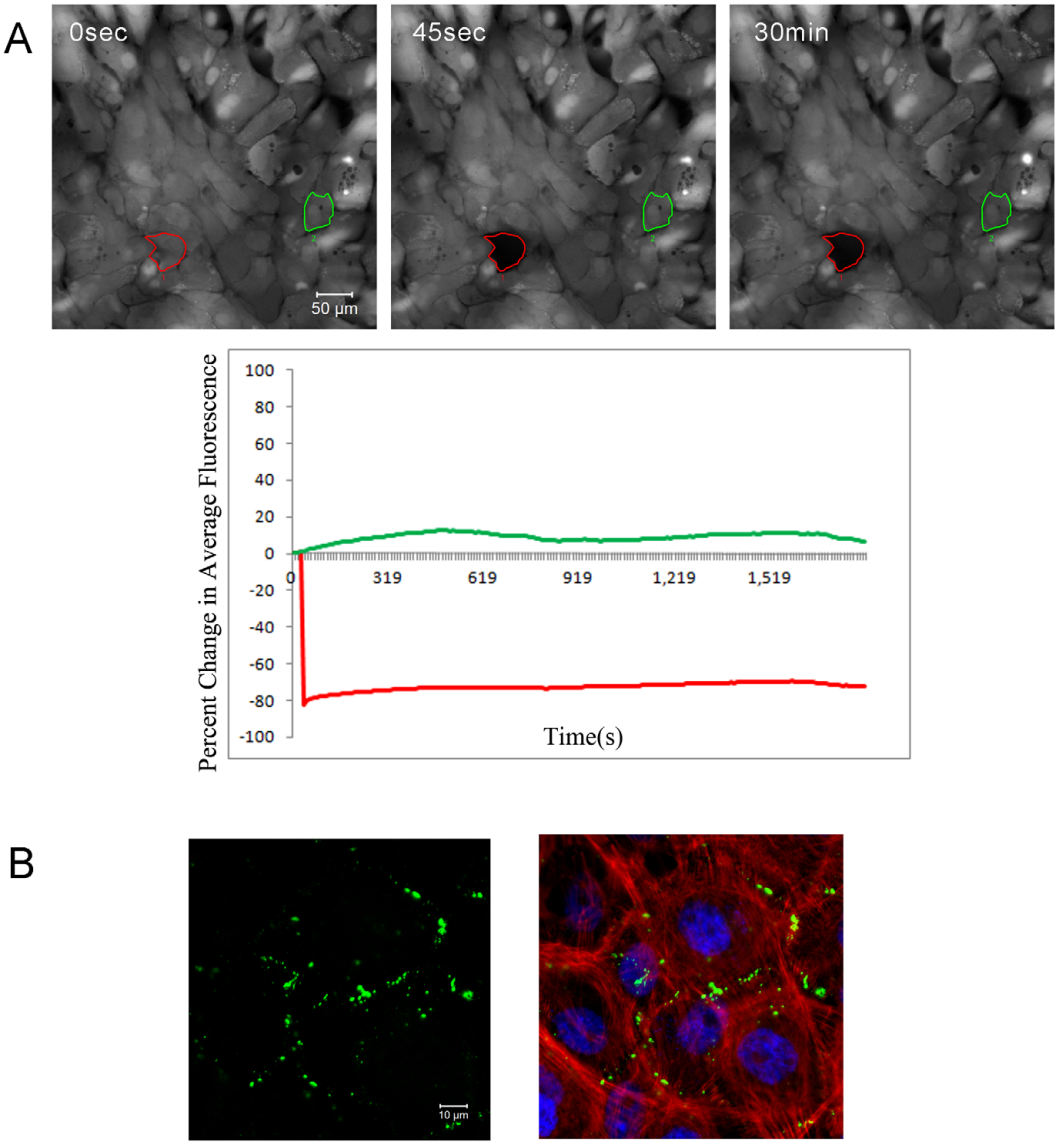
Epithelial cell communication depends on connexin/hemichannels. **A.** FRAP was performed with a cocktail of gap junction/hemichannel inhibitors. Cells were incubated in Carboxyfluorescein diacetate and individual cells were bleached with repeated laser iterations. Representative images of a typical FRAP experiment are shown, with circles denoting the bleached cell (red) and unbleached control cell (green). Recovery of cells over time is graphed as percent change in average fluorescence. Data represent a minimum of 4 independent experiments. **B.** Localization of Connexin 43 (green) along epithelial membrane (left panel) and counter-stained for F-actin with rhodamine phalloidin. Nuclei were stained with To-Pro-3 (blue) (right panel). Data represent a minimum of 3 independent experiments.

The aforementioned results were similar to those performed on corneal epithelial cells where Ca^2+^ mobilization was not inhibited after treatment with gap junction inhibitors applied singularly (1-heptanol, CdCl_2_ or GA) [11]. When parallel experiments were performed with a cocktail of inhibitors, cell communication between epithelial cells was impaired. To further examine this inhibition, fluorescence recovery after photobleaching (FRAP) experiments were performed in the presence of a cocktail of inhibitors. Epithelial cells were incubated in the presence of the cocktail inhibitor, incubated with 5-carboxyfluorescein diacetate (CFDA), photobleached and followed over time. The bleached epithelial cells were compared to unbleached cells in the same field. In the presence of the inhibitor cocktail recovery was negligible (Fig. 3A), but in the absence of inhibitors there was a 72% recovery over time (data not shown). The minimal decrease in fluorescence over time in the control unbleached cells was used as a basis for comparison. In addition, the typical punctate pattern along the cell membrane was detected when epithelial cells were immunostained with an antibody directed against Cx43 (Fig. 3B). Together these results suggest the presence of hemichannels in both neuronal and epithelial cells.

### Communication between Epithelial and Neuronal Cells Occurs in Co-cultures

**Figure 4:**
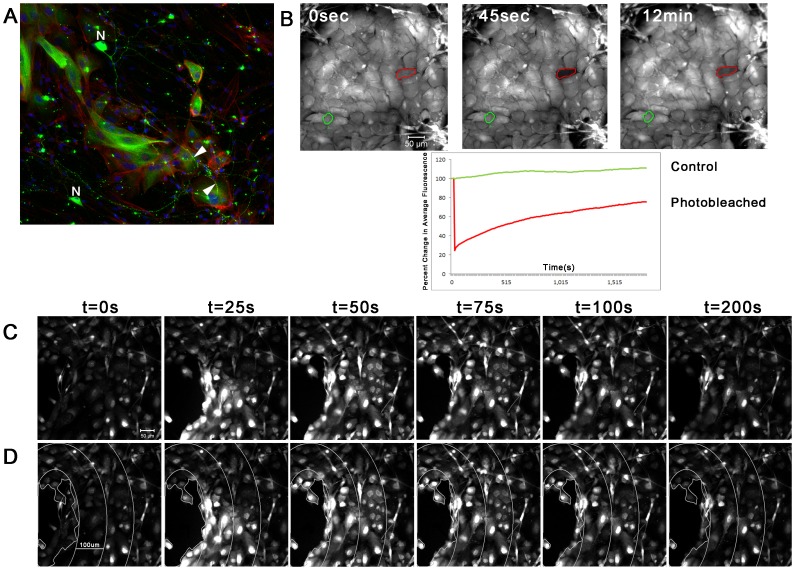
Communication between epithelial and neuronal cells in co-cultures. **A.** Neuronal cells (N) stained with β-III-tubulin (green) showed neuronal processes around and over epithelial cells (arrows). Cells were counter-stained for F-actin filaments (red) (rhodamine phalloidin) and nuclei (blue) (To-Pro-3). **B.** FRAP analysis shows rapid refill of epithelial cells (bleached cells -red) in co-cultures compared to control unbleached cells (green). **C.** Mobilization of Ca^2+^ wave after injury to epithelial cells. A series of images from a representative experiment is shown. **D.** Schematic showing concentric circles 100 µm apart radiating from wound site to determine distance of wave mobilization. The data are representative of a minimum of 4 independent experiments.

We developed a co-culture model to determine communication between epithelial and neuronal cells. Neurons and glia were allowed to adhere and epithelial cells were added once the neurons began to sprout processes. Three days after the addition of epithelial cells, the neuronal cells displayed a latticework of neuronal processes that passed over and between epithelial cells. The neuronal cells stained positively for β-tubulin (Fig. 4A) and the neurons extended over the epithelial cells. The epithelial cells stained diffusely for β-tubulin, which was reported in retinal pigment epithelial cells [44]. To determine if the co-cultures promoted communication, FRAP experiments were performed on epithelial cells in the co-cultures. The presence of the 2 cell types in contact with each other enhanced the rate of refill when compared to that of epithelial cells alone (Fig. 4B).

To determine mobilization of the Ca^2+^ wave after injury, the epithelial cells in the co-culture were injured using a pulled microcapillary pipette and the cultures were imaged every 786 msec for 8 min. The Ca^2+^ wave rapidly mobilized from the epithelial cells to adjacent neuronal cells (Fig. 4C). After each experiment concentric circles of 100 µm were overlayed with the initial point considered to be the wound site and the percent of responders determined for each distance to determine the duration of the wave (Fig. 4D, Table 2). The Ca^2+^ wave propagated 300 µm from the wound site without attenuation in the epithelial cells. After 400 µm over 60% of the cells continued to respond. A similar response was detected in the neuronal cells with over 70% responding after 400 µm (Table 2). To determine if communication depended on the release of nucleotides, the cultures were preincubated with Apyrase and the percent of epithelial responders decreased dramatically after 200 µm, while the percent of neuronal responders decreased by over 50% in the first 300 µm. The glial response also decreased correspondingly. We also evaluated the response to a cocktail of cell-junction inhibitors in the co-cultures and while the response was attenuated in the epithelial cells, the percent of epithelial responders was significantly greater than the neuronal responders (Table 2).

**Table 2:** Communication between epithelial and neuronal cells in co-cultures.

Distance(µm)		100	200	300	400
Control	Epithelium	100.0	100.0	100.0	66.7
	Neuron	100.0	100.0	100.0	75.0
	Glia	84.6	88.9	78.9	24.1
Apyrase	Epithelium	100.0	81.4	30.0	36.4
	Neuron	-	100.0	37.5	12.5
	Glia	100.0	38.9	3.4	3.7
Ca^2+^ - free	Epithelium	29.4	50.0	43.3	5.3
	Neuron	-	100.0	57.1	20.0
	Glia	0.0	0.0	11.1	2.6
Gap junctionInhibitors	Epithelium	75.0	33.3	10.7	0.0
	Neuron	0.0	16.7	0.0	0.0
	Glia	0.0	0.0	0.0	0.0
		**Percentage of Cells Responding**			

To differentiate between the roles of P2Y and P2X receptors, the co-cultures were incubated in HEPES Ca^2+^-free buffer containing ethylene glycol tetraacetic acid (EGTA). This Ca^2+^ chelator minimizes the contribution by the P2X receptors, which facilitate the recruitment of Ca^2+^ from the external source. In these co-cultures, the mobilization of the Ca^2+^ wave, shown as the percentage of cells responding, was reduced after 200 µm in epithelial cells and neurons when compared with the control (Table 2). These data indicate that P2X receptor(s) do play a role in cell communication following injury by promoting Ca^2+^ mobilization.

### Role of Secreted Epithelial and Neurotrophic Factors

Because the injury response in the co-culture system was complex and dissecting the role of each cell type was difficult, we evaluated the change in response to wound media of one cell type by the second cell type. Consequently, the following experiments were performed by wounding either the epithelial cells or the neuronal cells and evaluating the response of the wound media on the corresponding cells. Previously, we demonstrated that epithelial wound media added to epithelial cells elicited a Ca^2+^ mobilization that was attenuated when incubated with Apyrase II [7]. Therefore, we asked if the wound media from epithelial cells had a similar effect on neuronal cells. When epithelial wound media was added to neuronal cultures incubated in HEPES buffer containing fluo-3AM, there was a rapid Ca^2+^ mobilization that was reduced when the epithelial media was incubated with Apyrase II (1 U/mL) prior to addition to neuronal cells. Eighty percent of the neuronal cells responded to epithelial wound media, which was reduced to 10% when the media was pre-incubated with Apyrase (Student’s t-test, p<0.02). These experiments indicate that nucleotides released from injured epithelia are responsible for P2 activation of sensory neurons.

The reverse set of experiments was performed to determine the role of the neuronal wound media on epithelial cells. From the co-culture experiments, we detected the initial rapid response and a second prolonged response that was similar to Ca^2+^ mobilization induced by glutamate [45], [46]. Therefore, we asked if glutamate was present in the neuronal wound media and detected levels of 13.5 µM. Glutamate binds to either metabotropic G protein-coupled-receptors of the mGluR family or to the ionotropic receptors: α-amino-3-hydroxy-5-methyl-4-isoxazolepropionic acid receptor (AMPA), N-methyl-D-aspartate (NMDA), and kainate receptors, each named after their respective agonists. The NMDA receptor is a ligand-gated, voltage-dependent receptor that requires co-activation by both glutamate and glycine, but may also be activated by NMDA in place of glutamate.

**Figure 5:**
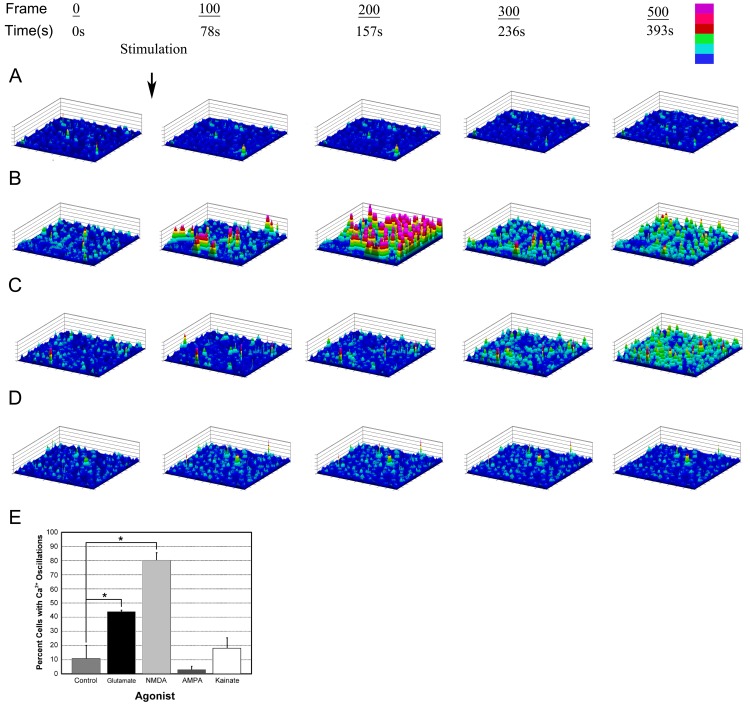
Components released from injury of neuronal cells on epithelial cells induce a distinct Ca^2+^ mobilization. Epithelial cultures were incubated with fluo3-AM and stimulated with the media harvested from primary neuronal cultures. Pseudo-colored images are displayed in 2.5D to show cell response over time using a six color intensity scale. Basal levels were obtained prior to stimulation. **A.** Conditioned neuronal media collected immediately before injury induces minimal mobilization over 600 frames. **B.** Neuronal media collected immediately after injury induces a rapid mobilization that is followed by a second elevation in Ca^2+^. **C.** Neuronal media collected immediately after injury and incubated with Apyrase did not cause an initial response but clusters of cells responded at later times. **D.** Neuronal media collected immediately after injury and incubated with Apyrase and NMDA inhibitors (LY233053, Ifenprodil, and MK-801) suppressed the cellular response. The data are representative of 3 runs for each condition from 6 independent cultures. **E.** Percent of epithelial cells that respond to glutamatergic agonists. Epithelial cells were incubated with fluo3-AM and stimulated with glutamatergic agonists (glutamate, NMDA, AMPA, and Kainate), and percent responders graphed. Glutamate and NMDA demonstrated significant increase in percent responders. Error bars represent s.e.m. Data represent a minimum of 3 runs. Significance was determined by Student’s t-test. *p<0.02.

To determine if the endogenous factors that were released into the media from injured neuronal cells and elicited a response were glutamate receptor agonists, experiments were conducted using NMDA inhibitors (Fig. 5). During each run of over 600 frames (0.79 s/frame), cells were exposed to control conditioned neuronal media, neuronal wound media, wound media incubated with Apyrase, or wound media incubated with Apyrase and inhibitors of NMDA receptors (Fig. 5). Incubation with conditioned media did not cause a change over the duration of the experiment (Fig. 5A). In the presence of neuronal wound media, there was an initial rapid mobilization that was followed by a sustained response in epithelial cell clusters (Fig. 5B). This response was similar to that of wounding neuronal cells in the co-culture experiments and imaging epithelial cells. Incubation of the neuronal wound media with Apyrase resulted in attenuation of the rapid initial response, however the delayed response between epithelial cells continued (Fig. 5C). In contrast, when the cells were stimulated with neuronal wound media incubated with both Apyrase and a cocktail of NMDA inhibitors (LY233053, Ifenprodil, and MK-801, a non competitive antagonist) the early and late responses were abrogated (Fig. 5D).

To verify if glutamate receptor agonists mediate epithelial communication, epithelial cultures were incubated in fluo3-AM and their response to specific agonists was evaluated. All incubations were performed in the presence of 10 µM glycine. There was a significant increase in the percent of cells that displayed intracellular oscillations in response to either glutamate or NMDA compared to HEPES buffer (control) (p<0.05) (Fig. 5E). Only a minimal response was detected when Kainate or AMPA was added.

### Epithelial Cells Express NMDA Receptors

**Figure 6:**
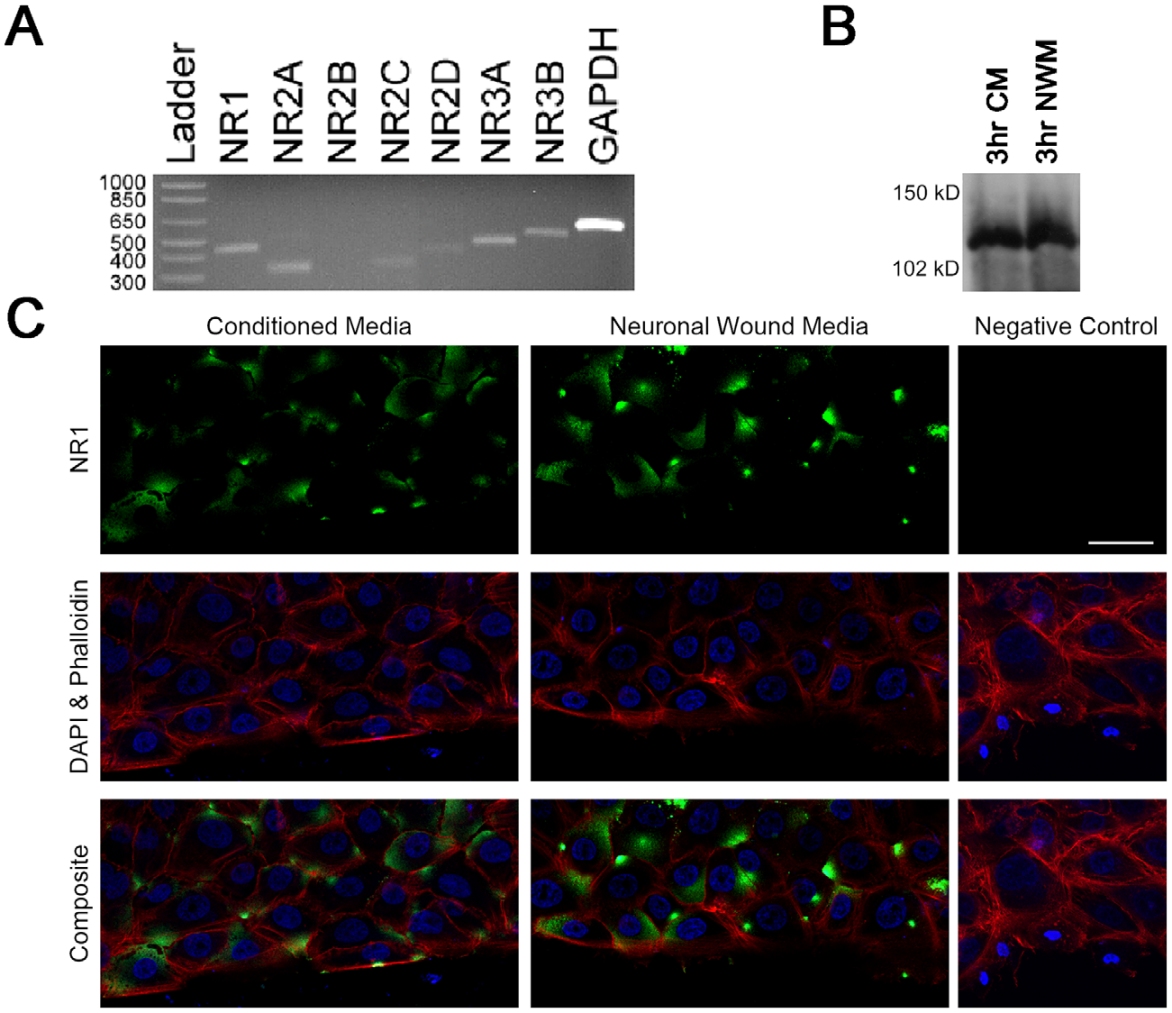
Localization and expression of NMDA receptors in epithelial cells. **A.** RT-PCR of epithelial extracts revealed the expression of NMDA receptors. **B.** Expression of NR1 by immunoblot analysis after treatment of cells with neuronal wound media (NWM) compared to treatment with conditioned media (CM). **C.** Confluent cultures were incubated overnight in medium lacking growth factors. Each slide was placed on a heated microscope stage, wounded, and incubated in an environmental chamber at 37°C and 5% CO_2_ for 20 h. Immunolocalization of NR1 after injury to corneal epithelial cells and exposure to conditioned media or neuronal wound media. Images were collected as tiles for cells incubated with conditioned and neuronal media, along with a representative tile from the negative control. Images are shown as a single optical slice **(**10 µm**)**. Scale bar –50 µm.

The response to neuronal wound media by epithelial cells and the lack of response in the presence of inhibitors prompted us to examine the expression and localization of glutamate receptors in epithelial cells. RT-PCR was performed using primers for human NMDA receptors and product identities were verified by sequencing analysis. NMDA receptor subunit gene products for NR1, NR2A, NR2C, NR2D, NR3A and NR3B were detected (Fig. 6A). When epithelial cells were exposed to either neuronal or conditioned media, immunoblots stained with an antibody directed against NR1, showed expression of the receptor indicating that the factors secreted from cells during culture and from injury cause expression of the NR1 (Fig. 6B).

We then asked if there were more subtle changes in NR1 in response to neuronal wound media or conditioned media that could be detected using confocal microscopy. To determine the localization of NR1 in wounded epithelial cultures exposed to neuronal wound media or conditioned media, confocal imaging was performed and tiles of contiguous areas along the wound margin and back from the wound were collected so that large areas could be compared [29] (Fig. 6C). The receptor was detected along the cell membrane and was present as punctate foci at points between cells. In further corroboration of these results, we asked if the neuronal wound media elicited a distinct phosphorylation of extracellular signal-regulated kinases (ERK) compared to that of epithelial wound media. Previously, we demonstrated that epithelial wound media or nucleotides induced an increase in pERK after 2 min that decreased significantly by 15 min [29], [47]. In contrast when neuronal wound media was added to epithelial cell cultures there was an increase in pERK after 15 min compared to pERK levels in conditioned media (Fig. S1).

### Neuronal Trophic Factors Mediate Cell Migration

**Figure 7:**
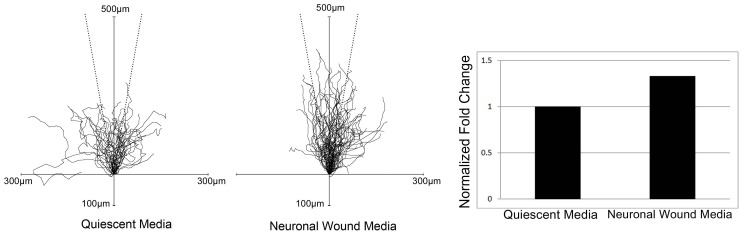
Neuronal wound media enhances directed cell migration. Confluent cultures were incubated overnight in medium lacking growth factors. Each slide was placed on a heated microscope stage, wounded, and incubated in an environmental chamber at 37°C and 5% CO_2_ for 20 h. The wounds were demarcated, and contiguous regions were tiled and imaged every 20 min. Cell migration was tracked using proprietary software (Zeiss) and placed on an axis where all cell paths start at the origin. An angle of 10 degrees on either side of the vertical axis (dotted line) was used to determine directed migration of epithelial cells over time in the different conditions. The number of cells migrating towards the opposite wound edge within 10 degrees of the vertical axis was normalized to 1 for those in the quiescent media condition using ImageJ. Data represent a minimum of 3 independent experiments.

In the next set of experiments, we asked if the NMDA receptor mediated cell migration or wound repair. Scratch wound assays were performed and neuronal wound media or control media was added. Images were taken every 20 min in an environmental chamber over a period of 24 hours. Cell migration rate and directed migration were evaluated. While there was a trend toward an increase in the rate of wound closure in the first six hours after injury, it was not significant. We then asked if there was a difference in directed migration or the path that the cells took in the presence or absence of the neuronal media. Image analysis of individual cells after injury demonstrated that the neuronal wound media generated a more focused, directed migration toward the other wound margin compared to control media (Fig. 7). A graph showing the track of cells and lines drawn 10 degrees on either side of the vertical demonstrated that neuronal media facilitated directed migration compared to control. Together, these data indicate that communication does exist between neuronal and epithelial cells that requires the activation of both purinergic and NMDA receptors.

## Discussion

The manuscript demonstrates that epithelial cells and neuronal cells use multiple receptor systems to communicate with each other in response to an injury. Both cell types release ATP with injury and possess a repertoire of P2Y and P2X receptors that may facilitate the response to pain, inflammation and repair [29], [48]. We speculate that ATP is released first from epithelial cells and is followed by the release of ATP and glutamate from the neuronal processes that activate both purinergic and NMDA receptors on epithelial cells. The latter explains both the rapid onset and a second set of responses that occurs in cell clusters. The fast-rising first phase is mediated by purinergic receptors while the slower second phase seen in epithelial cell clusters is inhibited by a cocktail of NMDA inhibitors. This reciprocating response between epithelial cells and neurons provides an alternative explanation of how the corneal epithelium and nerves are thought to respond to an injury.

Corneal innervation and resulting pathologies associated with denervation have been subjects of study for an extended period of time. The corneal epithelium has been used as a model for wound healing and matrix remodeling [1], [49], [50] and the release of nucleotides [6]–[8], [14], [18], [26]. Dissociated sensory neuronal cultures have been shown to have similar Ca^2+^ depolarization properties as in vivo studies [51]. Previously, investigators have examined the effects of neuropathy and shown that denervation of the cornea, whether it be due to causes such as infection, diabetes, or surgery, results in a reduction in viability of the corneal epithelium and its ability to repair damage. Moreover, there has been a dearth of studies examining the synergistic relationship between epithelial cells and neuronal processes during the initial wound response.

### Communication between Epithelia and Sensory Neurons

We developed a co-culture system to study the response between neuronal processes and epithelial cells in a controlled environment. Previous co-culture models have been developed to study the role of secreted products on proliferation compared to substance P [52]. In that model, the two populations of cells were not in direct contact with each other, but in our co-culture model, the trigeminal neurons and epithelial cells were in direct contact with each other facilitating the study of communication between them. Neurons and glia were allowed to adhere and sprout processes before the epithelial cells were plated. The primary trigeminal neuronal cultures exhibited the standard pseudo-unipolar morphology with a single axon extending from the soma [53]. The sensory fibers produced a complex network of processes throughout the culture. In addition, the somata were generally spherical in shape with a diameter of approximately 20–30 µm [54]. The primary cultures of neurons expressed P2Y_1,2,4,6 and 12_ and all known P2X receptors that were detected in the intact trigeminal neurons. The P2Y_6_ receptor was overexpressed and it is possible that the neurons are in a “pseudo-wounded” state [55]. This upregulation is supported by the observation that UDP caused the greatest number of neurons to respond at the lower concentrations and elicited the greatest relative fluorescence (165%). These are consistent with those of Ceruti et al [42], who demonstrated that neurons responded to UDP and bradykinin.

In previous wound studies, epithelial cells have been shown to display a Ca^2+^ wave that is generated at the wound site and crosses acellular regions, indicating a diffusible compound that was identified as ATP [11]. External applications of nucleotides corroborated these findings and we have shown that the P2Y_2_ receptor is critical to the wound response and cell migration in epithelial cells [6], [7], [28], [29]. In sensory neurons, the application of nucleotides has been shown to cause depolarization and activation of purinergic receptors [56]. After an intradermal application of ATP was shown to cause pain [57], the activation of P2X_2_ and P2X_3_ receptors initiating pain transmission became a focus of sensory afferent purinergic signaling [58]. Because we have shown that the trigeminal neurons express both the P2X_2_ and P2X_3_ receptors and that the corneal architecture includes a high density of afferent processes interspersed between epithelial cells, we hypothesized that mobilization of a Ca^2+^ wave from the epithelial cells to the neurons initiates depolarization. Our injury experiments demonstrated that the Ca^2+^ wave extended from epithelial cells to neurons and that 66% to 75% of the cells, respectively, responded at distances greater than 300 µm. When the co-cultures were incubated in HEPES buffer containing Apyrase, the number of epithelial cells and neurons responding at 300 µm was reduced to 30%.

Previous investigators have debated whether purinergic receptor responses are enhanced by the release of nucleotides from other cells and produce signal amplification through autocrine or paracrine signaling [11], [59], [60]. When primary neuronal cultures were incubated with a single inhibitor such as 1-heptanol or meclofenamic acid, there was no difference in fluorescence from increased intracellular Ca^2+^ compared to cultures incubated with the agonist alone. These results were similar to that of epithelial cells incubated in the presence of a single inhibitor [11]. However, additional experiments have shown that cell refill was inhibited when cultures were incubated with a cocktail of inhibitors, suggesting that small molecules such as ATP, Ca^2+^ or IP_3_ could pass from cells that have activated purinergic receptors to adjacent cells [59], [61]. In addition, release of ATP through hemichannels could potentially extend the distance of the wound response in both neurons and epithelial cells beyond the diffusional limit of the nucleotides released during injury [11].

### Release of Factors from Neuronal Cells Initiates Oscillatory Response in Epithelial Cells

While epithelial cells respond to injury with a simple sharp response where the mobilization attenuates over distance, the neuronal cells elicit a rapid peak and an oscillatory response from the epithelial cells. Furthermore, the addition of nucleotides causes a rapid Ca^2+^ increase that returns to basal levels within 90 s and is attenuated with Apyrase [6], [7], [11]. However, when neuronal wound media was added to epithelial cells, a response of long duration (over 15 min) was detected. While the Apyrase-treated neuronal wound media attenuated the primary response, the secondary response remained, indicating that nucleotide activation of P2 receptors did not cause the oscillations. However, when the media was incubated with a cocktail of NMDA inhibitors, the response was attenuated, which was supported by the presence of glutamate in the neuronal wound media indicating that the epithelial cells were responding to an excitatory neurotransmitter [45]. Furthermore, NR1 was localized in epithelial cells after injury and exposure to neuronal wound media. Since the neuronal cultures contained both peptidergic and P2X_3_ receptor-non-peptidergic neurons, we speculate that the source of glutamate could be from the synaptic vesicles, which contain VGLUT glutamate transporters. Studies have shown that glutamate transporters are found in trigeminal neurons [62] and most recently in corneal epithelia [61]. Furthermore, we have preliminary data demonstrating the expression of both Glut1 and Glut2 mRNA. However, unlike other studies where glutamate caused synchronous influx of Ca^2+^ by activating Kainate receptors [58], we detected oscillations in response only to NMDA and glutamate. Similar responses may occur in other tissues as investigators have shown that glutamate stimulates NMDA receptors causing a release of ATP in retinal pigment epithelial cells [63], and that both glutamatergic and purinergic receptors mediate Ca^2+^ responses in astrocytes of the cerebellar molecular layer [64].

Our results demonstrate that there is a tightly controlled feedback mechanism between epithelial and neuronal cells after injury. The release of ATP and increase in intracellular Ca^2+^ levels by epithelial cells after injury appears to be followed by an increase in ATP and glutamate by neuronal cells. The glutamatergic-purinergic interactions may promote both the wound response and promote prolonged communication between cells. Our results may explain why epithelial cells in culture heal but display impaired wound healing in vivo, where neuropathies may exist and presence of neuronal factors is altered.

## Materials and Methods

### Ethics Statement

All experiments described were conducted in voluntary compliance with the ARVO Statement for the Use of Animals in Ophthalmic and Vision Research. The Boston University Institutional Animal Care and Use Committee (IACUC) approved this study.

### Culture of Epithelial Cells and Trigeminal Neurons

Human corneal limbal epithelial (HCLE) cells were cultured [28], [65]. Previous experiments comparing HCLEs to primary epithelial cells demonstrated that the Ca^2+^ mobilization assays and internalization of EGFR are similar [28], [29].

Neonatal Sprague-Dawley rats (Charles River Labs, Wilmington, MA, USA) were euthanized and decapitated. The scalp and skull were cut, the brain was removed, and the mater and connective tissue were separated using a blunt probe to expose the trigeminal ganglia and cranial nerve V. The connective tissue beneath the ganglion was cut and the posterior nerve was cut. The trigeminal nerve was removed from the foramen and maintained in a cold protease solution containing trypsin/EDTA, 1.5 mg/mL collagenase A and 0.05 mg/mL papain. The ganglia were digested for 1 h at 37°C on a nutator, triturated, and the dissociated cells were resuspended in neurobasal medium supplemented with B27, 0.1 mM glutamine, 100U penicillin, 100 µg/mL streptomycin, 0.5% Fungizone and 15 ng/mL nerve growth factor (NGF). The resuspension was plated on fetal bovine serum (FBS) coated #1.5 glass coverslips or tissue culture plastic and cells cultured in supplemented neurobasal medium (Invitrogen, Grand Island, NY, USA).

For co-culture experiments, neurons were harvested and plated on one-half of a 30 mm glass-bottom culture dish and allowed to settle for 24 h. Epithelial cells were plated on the opposite half and cultures were maintained.

### Calcium Imaging

Calcium imaging was performed on HCLEs, neuronal cultures and co-cultures [6], [18], [28]. Briefly, cells were transferred into either an HEPES-buffered saline solution or in a CaCl_2_-free HEPES-buffered solution for 20 min and loaded with the Ca^2+^ indicator dye fluo 3-AM (5 µM) solution containing 0.05% pluronic acid/DMSO in HEPES. Cells were washed to remove excess dye. The cells were imaged using a Zeiss Axiovert 100 M LSM 510 confocal microscope equipped with Argon and 2 HeNe lasers (Zeiss, Thornwood, NY, USA). All perturbations were conducted while cells were continuously scanned using a Harvard apparatus perfusion chamber. Cells were perfused with HEPES before stimulation or injury to establish a baseline fluorescence reading. Cells were stimulated with an agonist and washed with HEPES-buffered saline and dynamics were imaged every 0.789 s/frame for 8 min. Fluorescence measurements for neurons and glial cells were randomly chosen and the relative percent change in fluorescence was calculated.

For wounding experiments, cells were cultured on a glass-bottom dish, transferred into HEPES-buffered solution, loaded with fluo-3-AM, washed and mounted on the Zeiss stage. Wounds of 100 µm were made while the field was continuously scanned for 600 frames at 0.79 s/frame. To analyze the response, the field was divided into concentric regions radiating from the center of the wound. The total number of cells was divided by the number of responding cells within each zone to calculate the percentage of responders in each zone. This was averaged over a minimum of 3 independent runs.

### Immunohistochemistry and Confocal Imaging

Cultures were fixed in freshly made 4% paraformaldehyde in phosphate-buffered saline (PBS) at pH 7.2 for 20 min at room temperature and processed for indirect immunohistochemistry [29]. Briefly, cells were washed with PBS, permeabilized with 0.1% Triton X-100 and blocked with PBS containing 5% bovine serum albumin (BSA). Proteins were probed for using NR1 (1∶50, BD Pharmingen, San Diego, CA, USA), β-tubulin (Tuj1) (R&D Systems, Minneapolis, MN) GFAP (1∶50, Invitrogen, Carlsbad, CA) and Cx43 (1∶50, Santa Cruz Biotechnology, Santa Cruz, CA). The cells were incubated with 5% BSA in PBS containing antibody for 18 h at 4°C. After incubation with the appropriate primary antibody, cells were rinsed with PBS, blocked with 3% BSA in PBS and incubated with the appropriate secondary antibody conjugated to a fluorescent molecule (Jackson Immunoresearch, West Grove, PA, USA or Molecular Probes, Eugene, OR, USA) for 2 h at room temperature and counterstained with rhodamine phalloidin (5U) and To-Pro-3 (1 µM) for 45 min at room temperature. Confocal laser scanning microscopy was performed using either a Zeiss Axiovert 100 M, 200 M LSM 510 or LSM 700 as described [6], [7], [66]. Images were collected as tiles, allowing us to scan a larger region of contiguous cells. The instruments contain the following laser modules: diode (405 nm), Argon (488 nm), HeNeI (543 nm) and HeNeII (633 nm). The proprietary software was used for analysis.

### RNA Isolation, RT-PCR and Real-Time PCR Analysis

RNA was extracted from cells or tissue using TRIzol reagent, according to the manufacturer’s protocol (Invitrogen). mRNA transcripts were purified using an Oligotex kit (Qiagen, Valencia, CA, USA). First strand cDNA synthesis was performed using Moloney murine leukemia virus (MMLV)-reverse transcriptase (Invitrogen) and 50 pmol of Anchored Oligo-dT primer of purified mRNA according to the manufacturer’s protocol (Invitrogen). Qualitative PCR amplification of rat and human P2Y, P2X, connexin and NMDARs was performed using 50 pmol of primers generated from NCBI GenBank, sequences using Primer6 design tool. The Taq polymerase used was Eppendorf Taqmaster (Eppendorf AG, Hamburg, Germany) under the following conditions: 95°C for 10 min (95°C for 30 s, 60°C for 30 s, 72°C for 1 min)×30 cycles, 72°C for 10 additional min. PCR products were cloned into pCR2.1 vectors (Invitrogen), transformed into TOP10 chemically-competent *E.coli* (Invitrogen), and cultured on X-gal treated LB/Amp agar plates. White colonies were picked, grown in LB/Amp media overnight, and the plasmids purified using Mini-prep kits (Qiagen). Plasmid insert sequences were obtained from Seqwright (Houston, TX, USA) using standard T7 and M13 promoter-specific primers, and the results verified by BLAST analysis of the NCBI GenBank. Oligonucleotides were produced from NCBI GenBank transcripts using the Primer6 program, and were designed to have a T_M_ = 60°C. Primers used were: P2Y_1_ gctgcagaggttcatcttcc (sense), ctcgggacagtctccttctg (antisense); P2Y_2_ gtgaccactggccatttagc, gccaggaagtagagcacagg; P2Y_4_ aggttctgcaggacagagga, gagaaagcggacaaacttgc; P2Y_6_ cagttatggagcgggacaat, tagcaggccagtaaggctgt; P2Y_12_ cctgaagaccaccagaccat, ctcagcatgctcatcaagga; P2X_1_ ccagttggtggttctggtct, tgactctcgcaccacatagc; P2X_2_ ctgcctcctcaggctacaac, ctaaagcaagacgcctgtcc; P2X_3_ ggaccattgggatcatcaac, cacacccagccgatcttaat; P2X_4_ gatggctactgcgtctgtca, acctgagagagcctccttcc; P2X_5_ accaacctgatcgtgactcc, tttcctgccttgccattaac; P2X_6_ tgtgacaccagctcaagtcc, gcagctggaaggagtactgg; P2X_7_ caagacccaaaggtgtcgtt, catagcaatgaggcgtcaga; Cx26 caaaacccagaaggtccgta, aggcatagaattgggccttt; Cx30 cgaccagcatagggaaggta, tctctgggcctgtgttctct; Cx32ggctcaccaacaacacatagaa, agtaatccctaggaggcagagg; Cx36 ttcccgcttctacatcatcc, gcccaaggacatccaactta; Cx37 taggggtcatctccttggtg, aggagaagtggggtgtgatg; Cx40 ttggactgctgagaggtgtg, aggggttgacctctgaacct; Cx43 aacagtctgcctttcgctgt, gagcagccattgaagtaggc; Cx45 tttgtgtgcaacacagagca, gagtctcgaatggtcccaaa; NR1 gtgtggtttgagatgatggg, atcctcattgaactccacgc; NR2A gtccttctccgactgtgagc, atcatcacccagacagaggc; NR2C agagcttcctggacctaccc, cgctccagtcgtactcttcc; NR2D cttcctagtgaacccctccc, gatcttctttccgtgcttgc; NR3A tgggcatcttagtgaggacc, ctgctttctcggacagttcc; NR3B ggcactttaaggtgtggagc, ggcggagttgatactgaagc; and GAPDH tgccactcagaagactgtgg, tgtgagggagatgctcagtg.

### SDS PAGE and Western Blot Analysis

Cells were rinsed after treatment with cold PBS (pH 7.4), placed on ice, lysed in 10 mM Tris-HCl (pH 7.4) containing 1% Triton X-100, 0.5% Nonidet P-40, 150 mM NaCl, 1 mM phenylmethylsulfonyl fluoride (PMSF), and 1 mM sodium orthovanadate (Na_3_VO_4_), and sheared by passing through a 20G needle. The precipitate was discarded after samples were centrifuged for 10 min at 12,000 rpm. Protein concentration was determined using a bicinchonic acid (BCA)-based method and equivalent amounts of protein (40 µg) from each sample were subjected to SDS-PAGE and transferred to a PolyScreen polyvinylidene difluoride (PVDF) membrane by the semi-dry method. Non-specific binding was blocked with 5% milk in a Tris buffer (7.25 mM Tris-HCl, 2.75 mM Tris-base, 100 mM NaCl, 0.1% Tween-20) according to Alamone Labs (Jerusalem, Israel). Membranes were probed with NR1 antibody (1∶1000), washed, and incubated with horseradish peroxidase-conjugated secondary antibody. The chemiluminescence enzymatic reaction was carried out (Denville Scientific, Inc., Metuchen, NJ, USA).

### Scratch Wound Assay

Epithelial cells were cultured on 8-well glass-bottom coverslip chambers and directed scratch wound assays were conducted as previously described [29]. Briefly, cells were allowed to achieve confluency and the media was replaced with unsupplemented media lacking epidermal growth factor (EGF) and bovine pituitary extract (BPE) 18–24 h before experimentation. Cells were treated with either unsupplemented media, media containing conditioned neuronal medium, or neuronal wound media and two linear wounds were made as previously described [29]. The cultures were placed on the Zeiss Axiovert 200 M LSM 510 laser scanning confocal microscope (Zeiss) in an environmental chamber maintained at 37°C and 5% CO_2_. Wounds were monitored every 20 min for 20 h using the multi-time module in the LSM software allowing for tiled images at multiple locations to be taken. LSM software was used to measure the wound at various time points, and percentage and rate of wound closure were calculated. LSM software was also used to track motility of single cells and calculate the total distance and direction traveled by each cell. Values were given as the mean +/− s.e.m. Statistical comparisons were made using Student’s t-test or ANOVA, followed by Tukey’s post hoc test.

## Supporting Information

Figure S1 **Neuronal wound media induces pERK.** HCLEs were cultured to confluence and stimulated with control media or neuronal wound media for 5 or 15 min. Lysates were probed with an antibody directed against pERK and reprobed with an antibody directed against ERK. pERK was normalized to ERK using ImageJ. Images are representative of 3 independent experiments.
(TIF)
