# Supplementary figures and images for: Communication between Corneal Epithelial Cells and Trigeminal Neurons Is Facilitated by Purinergic (P2) and Glutamatergic Receptors
Source: PLoS One. 2012 Sep 7;7(9):e44574. doi: 10.1371/journal.pone.0044574 (PMC3436752; doi:10.1371/journal.pone.0044574)

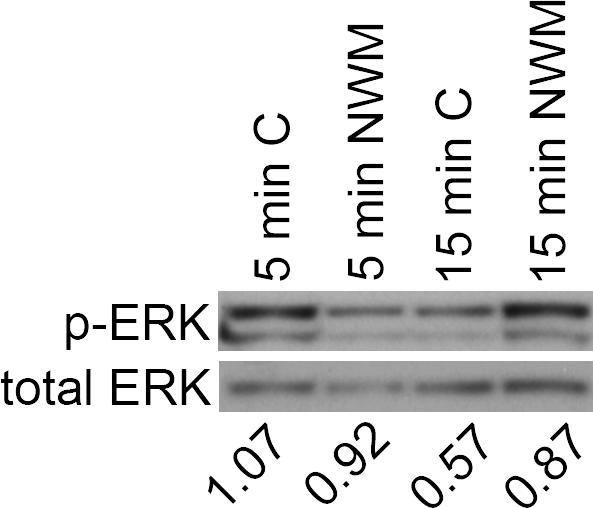

Supplement: Figure S1 — Neuronal wound media induces pERK. HCLEs were cultured to confluence and stimulated with control media or neuronal wound media for 5 or 15 min. Lysates were probed with an antibody directed against pERK and reprobed with an antibody directed against ERK. pERK was normalized to ERK using ImageJ. Images are representative of 3 independent experiments. (TIF) [file pone.0044574.s001.tif]
